# Abnormal Ribosome Biogenesis Partly Induced p53-Dependent Aortic Medial Smooth Muscle Cell Apoptosis and Oxidative Stress

**DOI:** 10.1155/2019/7064319

**Published:** 2019-05-09

**Authors:** Qi Wu, Junmou Hong, Zhiwei Wang, Junxia Hu, Ruoshi Chen, Zhipeng Hu, Bowen Li, Xiaoping Hu, Zhengpei Zhang, Yongle Ruan

**Affiliations:** ^1^Department of Cardiothoracic Surgery, Renmin Hospital of Wuhan University, Wuhan, Hubei Province, China; ^2^Department of Orthopedics, Renmin Hospital of Wuhan University, Wuhan, Hubei Province, China

## Abstract

Ribosome biogenesis is a crucial biological process related to cell proliferation, redox balance, and muscle contractility. Aortic smooth muscle cells (ASMCs) show inhibition of proliferation and apoptosis, along with high levels of oxidative stress in aortic dissection (AD). Theoretically, ribosome biogenesis should be enhanced in the ASMCs at its proliferative state but suppressed during apoptosis and oxidative stress. However, the exact status and role of ribosome biogenesis in AD are unknown. We therefore analyzed the expression levels of BOP1, a component of the PeBoW complex which is crucial to ribosome biogenesis, in AD patients and a murine AD model and its influence on the ASMCs. BOP1 was downregulated in the aortic tissues of AD patients compared to healthy donors. In addition, overexpression of BOP1 in human aortic smooth muscle cells (HASMCs) inhibited apoptosis and accumulation of p53 under hypoxic conditions, while knockdown of BOP1 decreased the protein synthesis rate and motility of HASMCs. The RNA polymerase I inhibitor cx-5461 induced apoptosis, ROS production, and proliferative inhibition in the HASMCs, which was partly attenuated by p53 knockout. Furthermore, cx-5461 aggravated the severity of AD *in vivo*, but a p53-/- background extended the life-span and lowered AD incidence in the mice. Taken together, decreased ribosome biogenesis in ASMCs resulting in p53-dependent proliferative inhibition, oxidative stress, and apoptosis is one of the underlying mechanisms of AD.

## 1. Introduction

According to the Global Burden Disease 2010 report, the death rate from aortic aneurysms (AA) and aortic dissection (AD) increased from 2.49 per 100,000 to 2.78 per 100,000 between 1990 and 2010, with higher frequencies among men [1, 2]. The common pathological basis of both is aortic media degeneration (AMD), which is characterized by a decrease in the number of aortic smooth muscle cells (ASMCs) [3] and matrix degeneration [4]. The phenotypic transformation of the ASMCs from the contractile to the proliferative form is involved in the process of AMD. Ribosome biogenesis is an essential process accompanying cell proliferation, and impaired ribosome biogenesis or function in mammalian cells leads to nuclear stress, which can cause cell cycle arrest, senescence, and apoptosis [5]. Studies show that atrophy of the skeletal muscle is partly due to impaired ribosome genesis [6, 7], while hypertrophy is associated with enhanced ribosome biogenesis [8, 9]. In the context of AMD, therefore, one can surmise that ribosome biogenesis is enhanced to aid ASMC proliferation. However, since decreased contractility is another significant change that occurs in the ASMCs during AMD, ribosome biogenesis ought to decrease in these cells. Therefore, the ribosomal status in ASMCs during AMD needs to be clarified.

Ribosome biogenesis is tightly regulated by the PeBoW complex, consisting of BOP1, Pes1, and WDR12, which is involved in 5.8S and 28S ribosomal RNA (rRNA) maturation. A dominant negative mutation in BOP1 has been associated with cell cycle arrest [10], whereas BOP1 overexpression in the liver and colorectal cancer cells increased their migration ability by activating the Wnt pathway [11, 12]. BOP1 has a short half-life due to the PEST motif [13], a common peptide motif in most “short-lived” proteins, which makes it the core modulator of the PeBoW complex [14]. Mutation in mouse BOP1 reduced the protein synthesis rate by nearly 75% [15]. In addition, blocking PeBoW complex function by Pes1 mutation induced p53 elevation [16], and the accumulation of total and phosphorylated p53 has been observed in the ASMCs during AMD [17].

In this study, we analyzed the potential role of the PeBoW complex in ASMC biology during AMD. We found a marked decrease in BOP1 levels in the aorta of AD patients compared to those of the brain dead donors, which was validated in a mouse model of AD. BOP1 knockdown in human ASMCs (HASMCs) slowed protein renewal, downregulated the contractile proteins *α*-SMA and MLC, inhibited wound healing ability, induced apoptosis and ROS production, and elevated p53 levels. On the other hand, overexpression of BOP1 slightly impaired the proliferation but inhibited apoptosis and ROS production and p53 accumulation under hypoxic conditions. Furthermore, the specific RNA polymerase I inhibitor cx-5461 reduced protein synthesis and induced apoptosis in the HASMCs. In the murine AD model as well, cx-5461 promoted the occurrence of AD, which was partly rescued in the p53-/- mice. Taken together, BOP1 regulates the redox balance, protein synthesis rate, and contractility and survival of ASMCs, and aberrant BOP1 expression is likely involved in AMD pathogenesis.

## 2. Materials and Methods

### 2.1. Clinical Samples

This study protocol was approved by the Clinical Research Ethics Committees of Renmin Hospital of Wuhan University of China. Informed written consent was obtained from all patients. Twenty-eight aortic media specimens were collected from acute type A thoracic AD patients who underwent emergency aortic replacement surgery between April 2017 and August 2017 and displayed no phenotypic characteristics of any of the known genetic cardiac disorders, such as Marfan's syndrome and Loeys-Dietz syndrome. In addition, 14 normal aorta samples were collected from brain dead patients who were registered as heart donors. All samples were carefully removed adventitia and intima. The clinical data of these patients are summarized in Table 1.

### 2.2. *β*-Aminopropionitrile Diet-Based Mouse AD Model and p53 Knockout Mouse

The ethical committee of the Renmin Hospital of Wuhan University approved the animal experiments, which were designed in accordance with the Wuhan Directive for Animal Research and the Current Guidelines for the Care and Use of Laboratory Animals published by the National Institutes of Health. A *β*-aminopropionitrile- (BAPN-) based mouse AD model was established according to a previous report [18]. Three-week-old male C57BL/6 mice were fed a regular diet (control group, *n* = 10) or BAPN diet containing 0.25% (*w*/*w*) BAPN (TCI, Japan, Cat# A0796) (BAPN group, *n* = 10). For ribosome biogenesis interference study *in vivo*, mice were injected intraperitoneally (*ip*) with cx-5461 in 50 mM NaH_2_PO_4_ (pH 4.5) at a dose of 50 mg/kg per day [19] with (cx-5461+BAPN group, *n* = 10) or without a concomitant BAPN diet (cx-5461 group, *n* = 10). The p53 knockout heterozygous (p53+/-) mice were purchased from Jackson Laboratory (Bar Harbor, ME) (stock no. 002101, C57BL/6.129S2-Trp53^tm1Tyj/J^) and crossed to obtain the p53-/- and p53+/+ littermates that were also placed on the BAPN diet to establish AD. All mice were monitored daily, and survival and death reasons were recorded. The aortic samples were collected either when the mice died or at the end of the 8-week study when they were decapitated after anesthetizing with 1% phenobarbital (Sigma, Cat# 57-33-0). The injured aortic tissues were resected and fixed in 4% formaldehyde, while the remaining tissues were stored in liquid nitrogen.

### 2.3. Histology, Immunohistochemistry/Immunofluorescence, and TUNEL Assay

Resected aortic samples from AD patients, donors, or animal model were fixed in 4% formaldehyde overnight, dehydrated, paraffin-embedded, and cut into 4 *μ*m thick sections. The tissue sections were stained with haematoxylin and eosin (HE), Masson, or elastin Van Giessen (EVG) stains as previously described [20–22]. The severity of broken elastin fibres was graded according to a previous report [23], in terms of the ratio of collagen (blue stained) to muscle fibres (red stained) outside the hematoma which was calculated by the Image J software based on Masson-stained images.

For immunohistochemistry (IHC)/immunofluorescence (IF), the sections were hydrated, heated for antigen retrieval, and treated with hydrogen peroxide to inactivate the endogenous peroxidase as per standard protocols. After blocking with 5% goat serum, the sections were incubated overnight with anti-BOP1 (1 : 200; Bioss Biotechnology, Cat# bs-12877R), anti-Ki67 (1 : 400; Cell Signaling Technology, Cat# 9449), and anti-p53 (1 : 50; Santa Cruz Biotechnology, Cat# sc-126) antibodies, along with the control IgG (1 : 100, Santa Cruz Biotechnology, Cat# sc-2025) at 4°C. The sections were then incubated with horseradish peroxidase- (HRP-) conjugated secondary antibody, washed with PBS, and stained with diaminobenzidine (DAB) (Sangon Biotech, Cat# A600140). The total and positively stained cells in 10 random fields of each aortic media section were counted at ×400 magnification, and the percentage of positive cells was calculated.

8-OHdG is the biomarker that indicated the DNA damage induced by ROS. The sections were processed as above and incubated with primary anti-8-OHdG antibody (1 : 200, Bioss, Cat# bs-1278R) and anti-*α*-SMA antibody (1 : 200, Servicebio, Cat# GB13044). The slides were then incubated with a secondary antibody conjugated with a fluorescent label (Cy3-conjugated goat anti-rabbit IgG (H+L) and FITC-conjugated goat anti-mouse IgG (H+L)) (1 : 200, Servicebio, Cat# GB21303 and GB22301) for 1 hour at room temperature and the cell nuclei counterstained with DAPI. Images were captured using a fluorescence microscope (BX63, Olympus, Japan).

The TUNEL assay was performed to detect apoptosis *in situ* using a commercially available kit (In Situ Cell Apoptosis Detection Kit, FITC, Sangon Biotech, Cat# E607178) according to the manufacturer's instructions [24]. Positive TUNEL staining was observed under a fluorescence microscope (TE2000U, Nikon, Tokyo, Japan) using the B-2A filter (450–490 nm excitation filter, 505 nm dichroic mirror, and 520 nm band pass filter) at ×400 magnification. The positively stained cells were counted in 10 random fields and the percentage apoptotic cells were calculated.

### 2.4. HASMC Culture and Genetic Manipulation

The HASMC line (ATCC® PCS-100-012™) was purchased from the China Centre for Type Culture Collection (CCTCC) and cultured in HASMC complete medium (Procell, Cat# CM-H081) at 37°C under 5% CO_2_ and 100% humidity. For serum-free and hypoxic treatment, the cells were cultured at 37°C in serum-free medium under 1% O_2_, 5% CO_2_, and 99% N_2_ in a humidified chamber (Binder, CB-210 hypoxia workstation). BOP1 knockdown in the HASMCs was established by RNA interference using BOP1 (si-BOP1: AUGGCAUGGUGUACAAUGAdTdT) and related scrambled (scr: UUCUCCGAACGUGUCACGUdTdT) siRNAs purchased from RiboBio. Briefly, 8 *μ*l of 20 *μ*M scr or si-BOP1 was diluted in 400 *μ*l Opti-MEM (Gibco, Cat# 31985062) and incubated with 5 *μ*l Lipofectamine 2000 (Invitrogen, Cat# 11668-027) for 25 min in room temperature. The mixture was then added to the HASMCs, and the cells were cultured for 6 h. To overexpress BOP1, HASMCs were transduced with adenovirus carrying BOP1 (Ad-BOP1; Vigene Bioscience Corporation, Cat# VH806931) or GFP (Ad-GFP; Vigene, Cat# CV1001).

### 2.5. Quantification of Protein Synthesis

The rate of protein synthesis was determined by detecting the amount of puromycin-labeled nascent polypeptides as previously described [25, 26]. The cells are cultured in puromycin-plus medium, which is incorporated into the elongating peptide chains and terminates mRNA translation, resulting in the release of the puromycin-labeled truncated peptides from the ribosome; the amount of which can be determined using antipuromycin antibodies to reflect the rate of protein synthesis. HASMCs transfected with si-BOP1/scr or treated with cx-5461 were incubated with 1 *μ*M puromycin for 40 min, harvested, and lysed. The cell lysates were suitably processed for western blotting, and the nascent proteins were detected using the antipuromycin antibody (1 : 1000; Merck Millipore, Cat# MABE343-AF488). To determine the total protein quantity in each sample, equal amounts of proteins (40 *μ*g) per sample were resolved by SDS-PAGE and the gels were stained with Coomassie blue.

### 2.6. Cell Viability Assays

HASMCs were seeded in 96-well plates at the density of 3 × 10^3^/190*μ*l/well and after overnight culture were treated with varying concentrations of cx-5461 (0-20 *μ*M) for 24 hours. For the viability assay, 10 *μ*l tetrazolium salt WST-8 (Cell Counting Kit-8; Keygen, Cat# KGA317) was added to each well (final volume 200 *μ*l), and the optical density at 450 nm was measured. The cell viability was calculated as OD450_treated_/OD450_control_ × 100%. To study the effect of p53 on cx-5461-induced cell death, HASMCs were pretreated with 10 *μ*M of the specific p53 inhibitor pifithrin-*α* (PFT*α*; Selleck, Cat# S2929) [27] for 12 h before cx-5461 treatment as described above. Similarly, to assess the influence of BOP1 on HASMC survival, the cells were transduced with Ad-BOP1 or Ad-GFP and 24 h later were resuspended and reseeded (0.8 × 10^3^/190*μ*l/well) in 96-well plates. The cell viability was determined daily for the next 5 days as described.

### 2.7. Western Blotting

Tissues or cells were washed with cold PBS and lysed in RIPA buffer (Beyotime, Cat# P0013K) supplemented with a protease inhibitor cocktail (Roche, Cat# 04693159001). The cell debris was removed by centrifugation (12000g, 4°C, 10 min) after ultrasonication, and the protein concentration of the cleared lysates was determined by BCA assay (Beyotime, Cat# P0010). Equal amounts of proteins (HASMCs, 20 *μ*g; human aortic tissue, 200 *μ*g; and mouse aortic tissue, 120 *μ*g) were resolved by 8%-12% SDS-PAGE and transferred onto polyvinylidene difluoride (PVDF) membranes (Merck Millipore, Cat# ISEQ00010). After blocking with 5% skimmed milk in PBS buffer, the membranes were incubated overnight with anti-BOP1 (1 : 100; Santa Cruz Biotechnology, Cat# sc-390672), anti-p53 (1:100; Santa Cruz Biotechnology, Cat# sc-126), anti-ACTA2 (smooth muscle cell actin, a-SMA) antibody (1 : 800, Proteintech, Cat# 14395-1-AP), antimyosin light chain-2 (MLC) antibody (1 : 500; Proteintech, Cat# 10906-1-AP), antipuromycin antibody (1 : 400; Merck Millipore, Cat# MABE343-AF488), antiactivated caspase 3 (1 : 1000; Cell Signaling Technology, Cat# 9664), and anti-GAPDH (1 : 5000; Proteintech, Cat# 60004-1-Ig) primary antibodies at 4°C. The membranes were washed and incubated with IRDye-800CW-conjugated goat anti-mouse IgG (1 : 20000; Li-cor, Cat# 926-32210) or goat anti-rabbit IgG (1 : 20000; Li-cor, Cat# 926-32211) secondary antibodies. The membranes were scanned by Odyssey (Li-cor Biosciences), and the grayscale value of each band was qualified by the paired software. At least 3 independent experiments were performed, except for the mouse aortic protein.

### 2.8. Wound Healing Assays

HASMCs were seeded in six-well plates and cultured till 90% confluence. After starving the cells for 12 h in serum-free medium, the confluent cell monolayer was gently scratched in a straight line with a 100 *μ*l pipette tip. The debris was removed and the edge of the scratch was smoothed with PBS washing. The gap was then monitored by phase contrast microscopy at the indicated time points. A minimum of three independent experiments was performed.

### 2.9. Cytometric Analysis of Cell Apoptosis

Apoptosis in the HASMCs was detected using the Annexin V-APC/7-AAD apoptosis detection kit (BD Biosciences, Cat# 561012). The cells were harvested and washed twice with PBS containing 5% FBS and resuspended in 500 *μ*l binding buffer provided in the kit. The cells were then incubated with 5 *μ*l Annexin V-APC and 5 *μ*l 7-AAD at room temperature for 15 min in the dark. The percentage of apoptotic cells was detected by flow cytometry using Cell Quest software (BD Biosciences, San Jose, CA, USA).

### 2.10. Detection of Reactive Oxygen Species (ROS)

Production of ROS was detected by 5 *μ*M dihydroethidium (DHE, Yeasen Biotech Co., Cat# 50102ES02). Briefly, HASMCs were pretreated with 10 *μ*M PFT*α* for 12 h and administrated with varying doses of cx-5461 for 24 h. After that, 5 *μ*M DHE was added in the medium and incubated at 37°C for 20 min. After incubation, HASMCs were washed with PBS, and fluorescence of DHE was detected using a confocal microscope. The ROS accumulation was also detected by DCFH-DA kit (Solarbio, Cat# CA1410). HASMCs were treated as stated above and stained by DCFH-DA working solution (10 *μ*M). Cellular fluorescence at excitation and emission frequencies of 488 nm and 525 nm, respectively, was measured using flow cytometry (BD FACS Calibur, USA).

### 2.11. Quantitative Real-Time PCR (qRT-PCR)

Total RNA was isolated by RNAiso Plus (Takara, Cat# 9109) according to the manufacturer's instructions. The concentration and purity of RNA were determined using ultraviolet spectrophotometry (Beckman Coulter, USA). The cDNA was synthesized using the RevertAid First Strand cDNA Synthesis Kit (Thermo Scientific, Cat# K1622) according to the manufacturer's instructions. RT-PCR analysis was performed using the SYBR Premix Ex Taq II (Takara, Cat# RR820A) in Biosystems 7500 Real-Time PCR Systems (ABI, USA). The primer sequences were as follows: BOP1 forward: 5′*-*GTGGGCTTCAACCCCTATGAG-3′, reverse: 5′-CCATGCGAGAGACCTTCTCC-3′; MLC forward: 5′-TTGGGCGAGTGAACGTGAAAA-3′, reverse: 5′-CCGAACGTAATCAGCCTTCAG-3′; *α*-SMA forward: 5′-AAAAGACAGCTACGTGGGTGA-3′, reverse: 5′-GCCATGTTCTATCGGGTACTTC-3′; and GAPDH forward: 5′-ACTTTGGTATCGTGGAAGGACTCAT-3′, reverse: 5′-GTTTTTCTAGACGGCAGGTCAGG-3′.

### 2.12. Statistical Analysis

Statistical analysis was performed using GraphPad Prism 5 software. Measurement data was presented as mean ± SD and compared using Student's *t-*test or *one-way ANOVA* test. Ranking data (elastin broken grading score) were analyzed by *Mann-Whitney test*, and the *chi-squared test* was used to compare incidence of aortic rupture between different groups. A *log-rank (Mantel-Cox) test* was used to compare Kaplan-Meier survival curves. *P* values < 0.05 were regarded as statistically significant.

## 3. Results

### 3.1. BOP1 Expression Is Decreased in ASMCs of AD Patients

**Figure 1:**
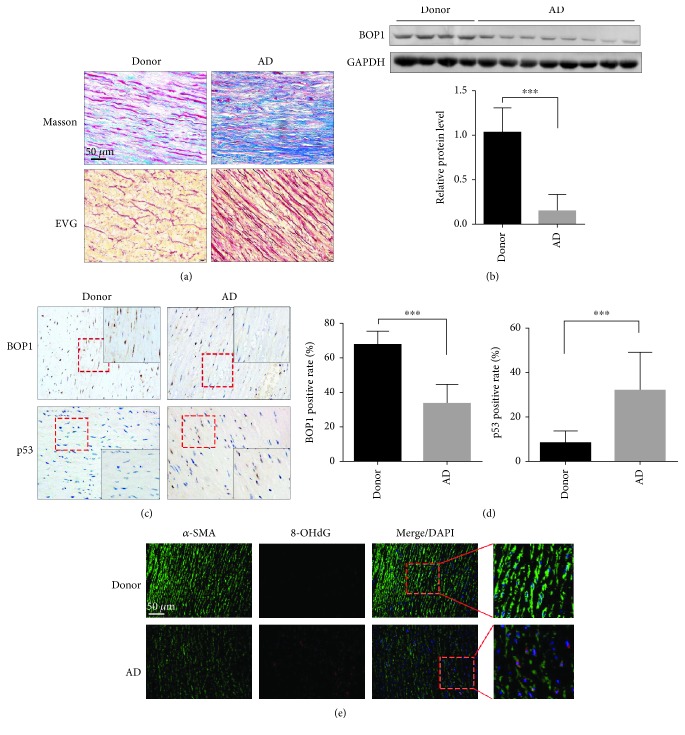
BOP1 expression is decreased in ASMCs of AD patients. (a) Images of Masson staining showed collagen (blue) and muscle fibre (red) in the aortic media derived from AD patients and donors (upper panel). Representative images of EVG staining indicated the broken elastic fibre in aortic samples derived from AD patients and donors (lower panel). (b) BOP1 protein expression in the aortic media of donors (*n* = 4) and AD patients (*n* = 8) was detected by western blotting, and the related expression level was detected by statistical analysis and shown. (c) Representative image of the aortic specimens stained by BOP1 and p53 by performing IHC. (d) The positive rate was detected by statistical analysis and shown. (e) The 8-hydroxy-2′-deoxyguanosine (8-OHdG) level in the aortic media tissues were detected by performing immunofluorescence and the representative images are shown. Scale bar 50 *μ*m. Data are presented as mean ± SD. ^∗∗∗^ *P* < 0.001 determined by Student's *t-*test.

The clinical data of 28 AD patients and 14 donors are summarized in Table 1, and significant differences were seen in terms of age and gender. The main features of AMD are loss of ASMCs, collagen accumulation, and fragmentation of elastic fibres. Masson staining showed an increase in the ratio of the collagen to muscle fibres in the aortic media of AD patients compared to that of donors (Figure 1(a), upper panel), while EVG staining indicated fragmented elastic fibres in the AD aortic samples (Figure 1(a), lower panel). BOP1 is the crucial component of PeBoW complex, which regulates rRNA processing, and due to its short half-life on account of the PEST motif, it might be indicative of rRNA maturation. A significant decrease was seen in the BOP1 protein levels in the aortic media of AD patients (*n* = 8) compared to those of the donors (*n* = 4) by western blotting (Figure 1(b)). Furthermore, BOP1 protein expression *in situ* was also downregulated in the ASMCs of AD patients (*n* = 28) compared to donors (*n* = 14) and largely localized to the nucleus (Figures 1(c) and 1(d)). Since ribosome biogenesis is closely related to p53, we further examined the *in situ* p53 expression and found significant elevation and nuclear accumulation (Figure 1(c)) in the aorta of AD patients (*n* = 28) compared to donors (*n* = 14) (Figures 1(c) and 1(d)). We also found accumulative ROS in the ASMCs of AD patient by detecting 8-OHdG (Figure 1(e)).

### 3.2. Overexpression of BOP1 Attenuated HASMC Apoptosis under Serum-Free and Hypoxic Conditions

**Figure 2:**
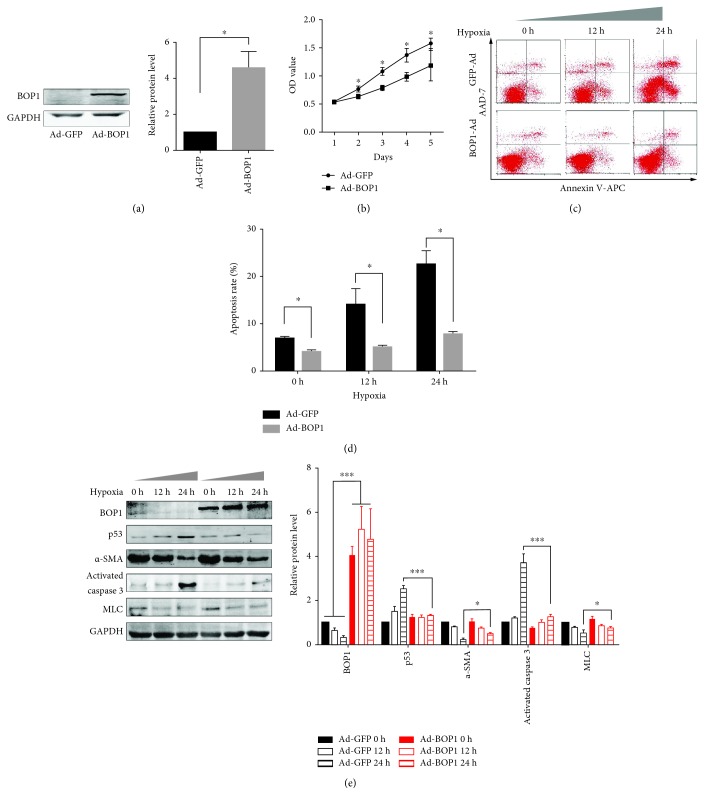
Overexpression of BOP1 attenuated HASMC apoptosis under serum-free and hypoxia condition. (a) The expression of BOP1 in HASMCs was detected after it had been infected with Ad-BOP1 or Ad-GFP for 24 h (left panel). Statistical analysis is shown (right panel). (b) HASMCs were infected with Ad-BOP1 or Ad-GFP for 24 h. The cells were suspended and reseeded in 96-well plates. CCK-8 assays were performed to assess the influence of BOP1 on HASMC proliferative ability. The growth curve is shown. (c) After being infected with Ad-BOP1 or Ad-GFP for 24 h, HASMCs were administrated in serum-free and hypoxia condition for the time shown. Apoptosis was detected by Annexin V-APC/7-AAD staining and flow cytometry followed. The representative images are shown. (d) The statistical analysis of apoptosis rate is shown. (e) Western blotting was performed to detect the BOP1, p53, *α*-SMA, activated caspase 3, and MLC expression. The representative image is shown (left panel). The statistical analysis is shown (right panel). Data are representative of three independent experiments and presented as mean ± SD. ^∗^ *P* < 0.05, ^∗∗^ *P* < 0.01, and ^∗∗∗^ *P* < 0.001 determined by *one-way ANOVA*.

HASMCs transduced with Ad-BOP1 showed significant elevation in BOP1 expression levels compared to the Ad-GFP-transduced cells (Figure 2(a)). Since BOP1 is overexpressed in various tumors, we tested its influence on HASMC growth by the CCK-8 assay. Surprisingly, however, overexpression of BOP1 inhibited cell proliferation (Figure 2(b)), although it reversed the time-dependent apoptosis induced in the HASMCs under serum-free and hypoxic conditions (Figures 2(c) and 2(d)). In addition, overexpression of BOP1 significantly alleviated the increased levels of proapoptotic proteins like activated caspase 3 and p53. In the control cells, hypoxia reduced BOP1 expression in a time-dependent manner (Figures 2(e) and 2(f)).

### 3.3. BOP1 Knockdown Impaired HASMC Protein Synthesis Rate and Motility

**Figure 3:**
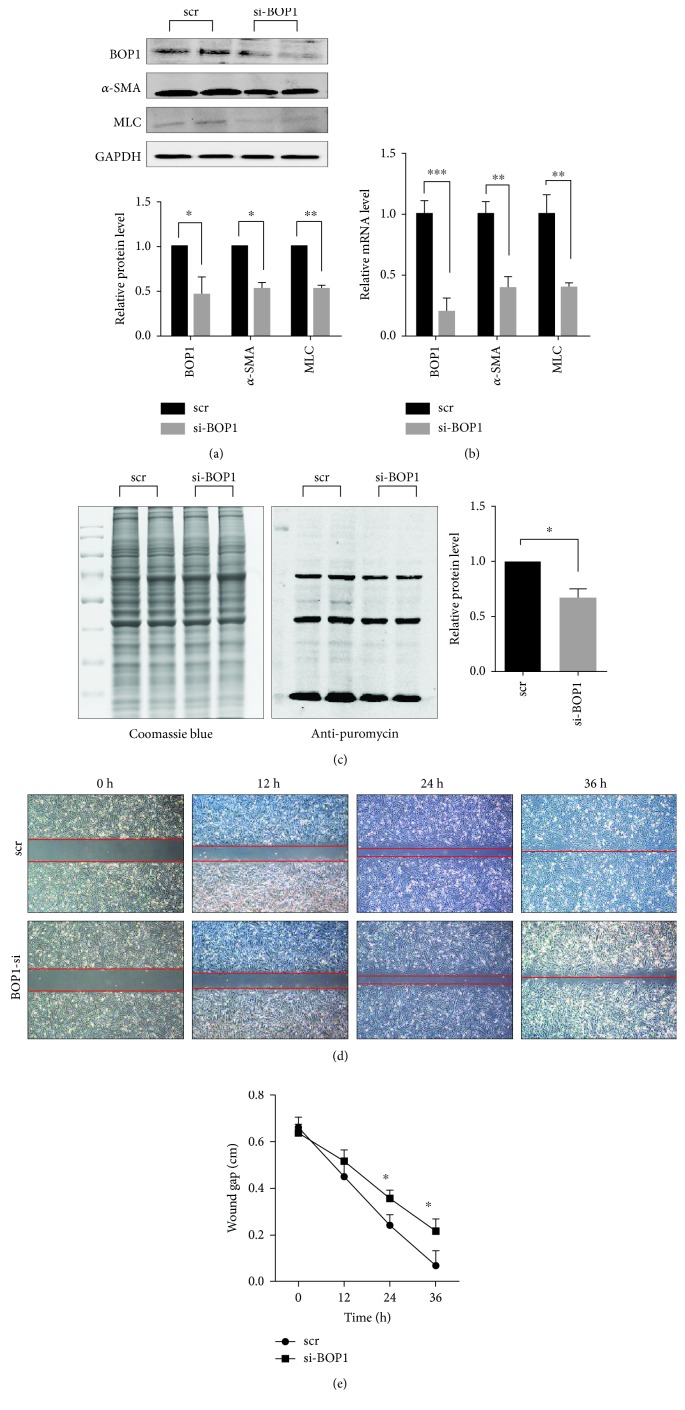
BOP1 knockdown impaired HASMC protein synthesis rate and motility. (a) After being transfected with scramble (scr) or BOP1 siRNA (si-BOP1) for 48 h, the expression of BOP1, *α*-SMA, and MLC was detected by western blotting. The expression levels were detected by statistical analysis and shown. (b) Real-time PCR was performed to detect the mRNA level of BOP1, *α*-SMA, and MLC in HASMCs after being transfected with si-BOP1 or scr for 48 h. (c) HASMCs were transfected with si-BOP or scr for 48 h and administrated with puromycin (1 *μ*g/ml) for 40 min to label the nascent protein. The equal amount of protein was electrophoresed and stained with Coomassie blue to indicate the total protein (left panel). Western blotting was performed to detect the nascent protein by using antipuromycin antibody (middle panel). The statistical analysis of nascent protein/total protein is shown (right panel). (d) Wound healing assay detected the mobility of HASMCs after being transfected with si-BOP1 for 48 h and photographed at the indicated time. The red dotted lines indicated the extent of scratches. (e) The extent of scratches was measured and detected by statistical analysis. Data are representative of three independent experiments and presented as mean ± SD. ^∗^ *P* < 0.05, ^∗∗^ *P* < 0.01, determined by Student's *t-*test.

To determine the role of BOP1 in HASMC motility, we examined the effect of altering BOP1 expression on the levels of *α*-SMA and MLC, which are associated with the contractility and motility of HASMCs. BOP1 knockdown decreased the levels of *α*-SMA and MLC in the HASMCs (Figures 3(a) and 3(b)). In addition, HASMC motility was also assessed by the *in vitro* wound healing assay, which showed significant inhibition of scratch recovery after BOP1 knockdown (Figures 3(d) and 3(e)). To determine the potential effect of BOP1 on the protein synthesis rate in HASMCs, we pretreated cells with puromycin to label the nascent peptides and detected them using the antipuromycin antibody. As shown in Figure 3(c), BOP1 knockdown significantly decreased protein synthesis rate in HASMCs.

### 3.4. cx-5461-Mediated Inhibition of RNA Polymerase I Affected Protein Synthesis and p53-Dependent Cell Apoptosis

**Figure 4:**
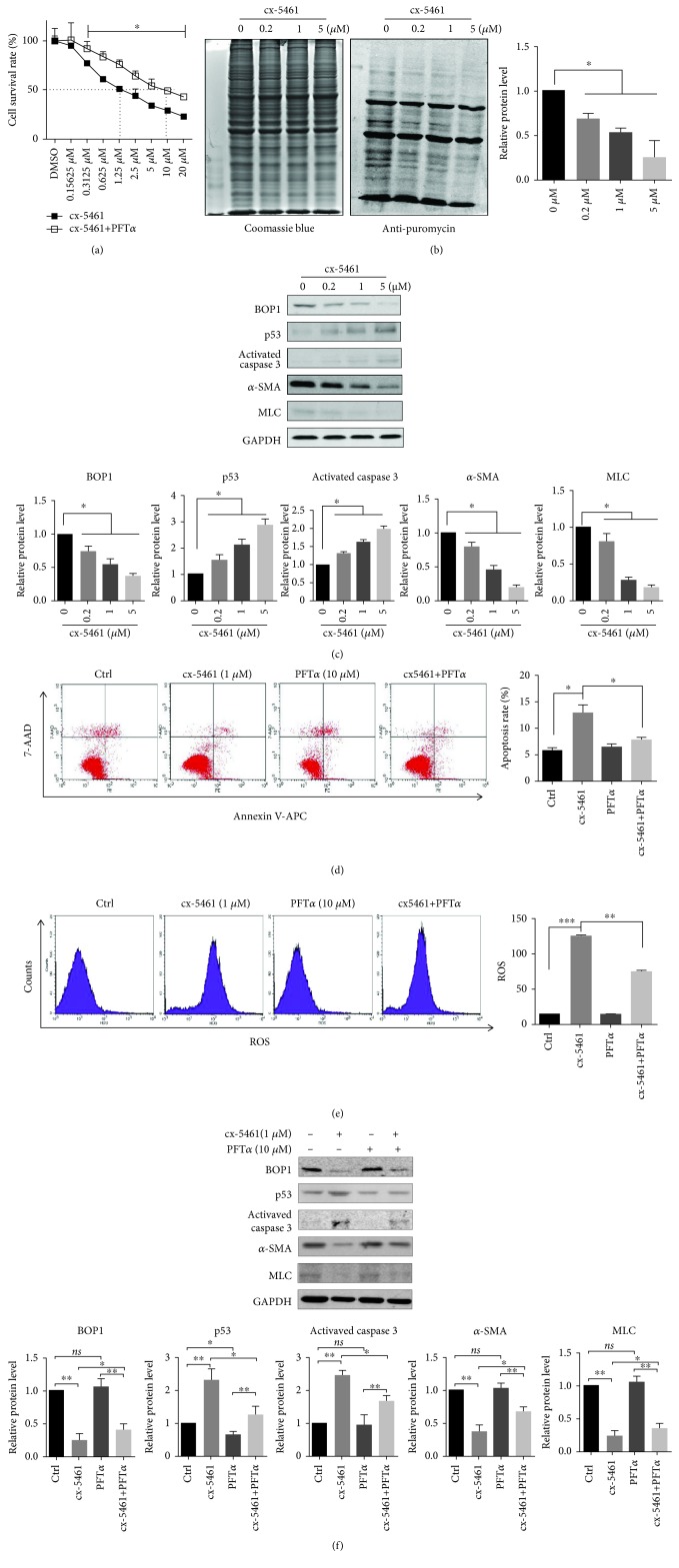
cx-5461 impaired protein synthesis and activated p53-dependent cell apoptosis. (a) HASMCs were pretreated with or without PFT*α* (10 *μ*M) for 12 h and then administrated with the indicated concentration of cx-5461 for 24 h. The cell survival rate was detected by CCK-8 assays. (b) HASMCs were treated with the indicated concentration of cx-5461 for 24 h and then administrated with puromycin (1 *μ*M) for 40 min. The total protein (Coomassie blue staining, left panel) and the nascent protein (antipuromycin, middle panel) were shown. The statistical analysis of nascent protein/total protein is shown (right panel). (c) HASMCs were administrated with the indicated concentration of cx-5461 for 24 h. Western blotting was performed to detect the BOP1, p53, activated caspase 3, *α*-SMA, and MLC expression. Representative images are shown (left panel), and the grayscale was measured and detected by statistical analysis (right panel). (d) HASMCs were pretreated with or without PFT*α* (10 *μ*M) for 12 h, followed by the treatment of cx-5461 (1 *μ*M). Apoptosis was assessed by Annexin V-APC/7-AAD staining and flow cytometry (left panel). The statistical analysis of apoptosis rate is shown (right panel). (e) ROS were detected by DCFH-DA and flow cytometry (left panel). The ROS level is shown (right panel). (f) HASMCs were pretreated with or without PFT*α* (10 *μ*M) for 12 h, followed by the treatment of cx-5461 (1 *μ*M). Western blotting was performed to detect the BOP1, p53, activated caspase 3, *α*-SMA, and MLC expression. Representative images are shown (left panel), and the grayscale was measured and detected by statistical analysis (right panel). Data are representative of three independent experiments and presented as mean ± SD; *ns*: no statistical significance; ^∗^ *P* < 0.05, ^∗∗^ *P* < 0.01, determined by *one-way ANOVA*.

To elucidate the association between ribosome biogenesis and apoptosis, HASMCs were treated with cx-5461, an inhibitor of RNA polymerase I. CCK-8 assay indicated significant cytotoxicity of cx-5461 in HASMCs (IC50 = 1 · 27 ± 0 · 19*μ*M), which was however attenuated when the cells were pretreated with the p53 inhibitor PFT*α* (IC50 = 9 · 66 ± 0 · 41*μ*M) (*P* < 0.001, Student's *t*-test; Figure 4(a)). In addition, cx-5461 also resulted in a dose-dependent reduction in nascent protein synthesis (Figure 4(b)), along with increased p53 and activated caspase 3 levels, and a dose-dependent decrease in the levels of BOP1, *α*-SMA, and MLC (Figure 4(c)). Furthermore, apoptosis and ROS production induced by cx-5461 in HASMCs were attenuated upon PFT*α* pretreatment (Figures 4(d) and 4(e); Fig.  S1). Consistent with this, p53 and activated caspase 3 protein levels also decreased in the PFT*α* pretreated cells. PFT*α* also partially reversed the cx-5461-induced decrease in BOP1, *α*-SMA, and MLC levels (Figure 4(f)).

### 3.5. Inhibition of RNA Polymerase I by cx-5461 Accelerated AD in Mice

**Figure 5:**
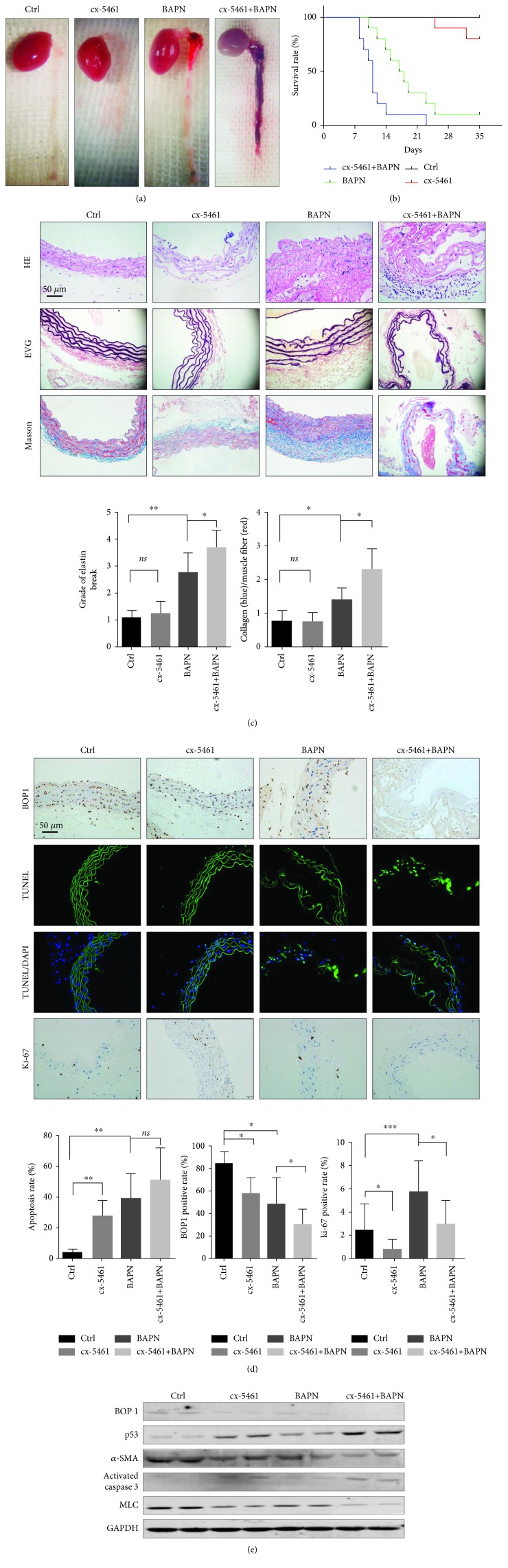
Inhibition of RNA polymerase I by cx-5461 accelerated AD in mice. (a) Representative images of gross aortic samples are shown. (b) The life-span of each mouse was recorded. Kaplan-Meier survival curve is shown. (c) Representative staining of aorta sections with HE, Masson, and EVG. Graphs show semiquantification of elastic fibre broken grade and collagen/muscle fibre ratio. (d) Representative images of the aortas performed with TUNEL assays, IHC staining with anti-BOP1 antibody and anti-ki-67 antibody. The positive rate is shown (right panels). (e) Western blotting was performed to detect the BOP1, p53, activated caspase 3, *α*-SMA, and MLC expression of the aortas. Data are presented as mean ± SD. ^∗^ *P* < 0.05, ^∗∗^ *P* < 0.01, and ^∗∗∗^ *P* < 0.001 determined by *one-way ANOVA*.

In order to elucidate the effects of ribosome dysfunction on AD, we established a murine AD model based on BAPN diet and treated the animals with cx-5461 (50 mg/kg/day). Mice in the cx-5461+BAPN group (*n* = 10) had an accelerated development and increased severity of AD and shorter life-span compared to the control group (*n* = 10) (Figure 5(b)). EVG staining showed a higher grade of elastin fibre breakdown (Figure 5(a)), while Masson staining revealed a higher collagen-to-muscle fibre ratio in the aortic tissues of the cx-5461+BAPN mice (Figure 5(c)). Mice fed with the BAPN diet also showed decreased BOP1 expression in their ASMCs, which declined further when treated with cx-5461. Furthermore, cx-5461 treatment further increased apoptosis and ROS production in the ASMCs of AD mice and reduced the AD-induced higher proliferative rates (Figure 5(d); Fig.  S2). Consistent with this, cx-5461 exacerbated the increase in activated caspase 3 and p53 levels and the decrease in *α*-SMA and MLC (Figure 5(e)).

### 3.6. Knocking Out p53 Reduced the Occurrence of AD in Mice

**Figure 6:**
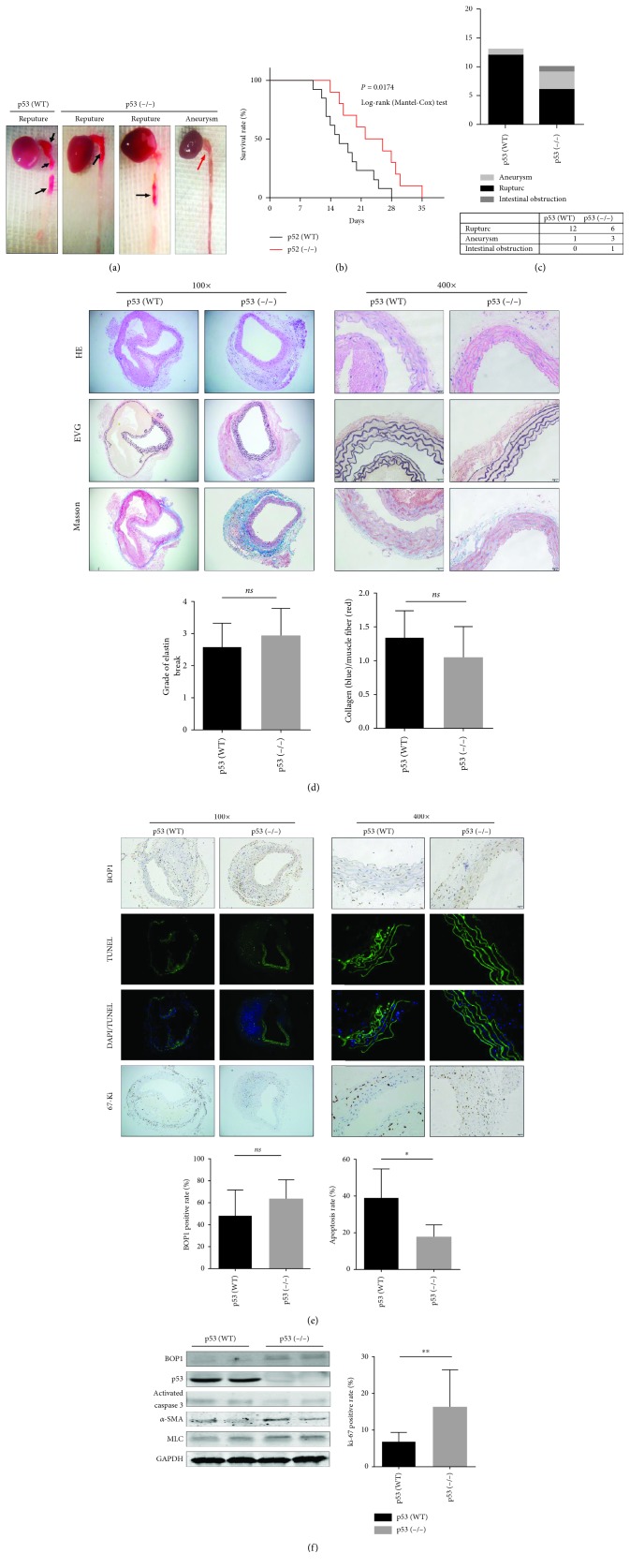
Knockout of p53 reduced the occurrence of AD in mice. (a) Representative images of gross aortic samples are shown. (b) Kaplan-Meier survival curve is shown. (c) The death reason is summarized and shown. (d) Representative staining of aorta sections with HE, Masson, and EVG. Graphs show semiquantification of elastic fibre broken grade and collagen/muscle fibre ratio. (e) Representative images of the aortas performed with TUNEL assays, IHC staining with anti-BOP1 antibody and anti-ki-67 antibody. The positive rate is shown (right panels). (f) Western blotting was performed to detect the BOP1, p53, activated caspase 3, *α*-SMA, and MLC expression of the aortas. Data are presented as mean ± SD; *ns*: no statistical significance; ^∗^ *P* < 0.05, ^∗∗^ *P* < 0.01, and ^∗∗∗^ *P* < 0.001 determined by *one-way ANOVA*.

Previous studies have shown that impaired rRNA transcription increases apoptosis in ASMCs, a phenomenon associated with p53 accumulation. Therefore, we established the AD model in p53-/- mice to explore its role in AD. The p53-/- AD mice (*n* = 10) had an extended life-span compared to the p53+/+ AD mice (*n* = 13) (Figure 6(b)). The representative images of the gross aorta are shown in Figure 6(a). All save one (12/13, 92.3%) p53+/+ AD mice died of aortic rupture, hemothorax, and major bleeding, while only 60% (6/10) of the p53-/- AD mice died of aortic rupture and the remaining 30% of aortic aneurysm and 10% of intestinal obstruction (Figure 6(c)). However, no significant differences were observed between the mice in terms of the severity of elastic breakdown and collagen-to-muscle fibre ratio (Figure 6(d)), and only a slight increase was seen in aortic BOP1 expression in the p53-/- AD mice (Figure 6(e)). However, knocking out p53 decreased apoptosis and ROS production and increased the proliferative rate (Ki67^+^ cells) among the ASMCs in the AD model (Figure 6(e); Fig.  S3). Consistent with the results of TUNEL assay, activated caspase 3 levels were decreased in the aortic samples of p53-/- AD mice (Figure 6(f)).

**Figure 7:**
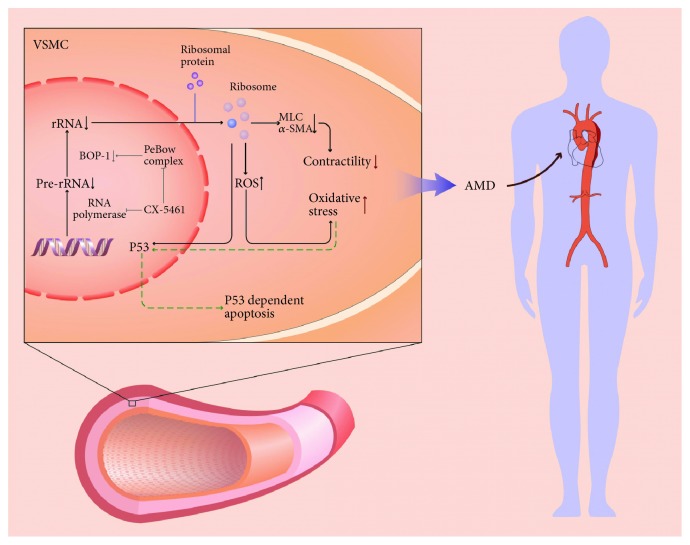
Schematic diagram of the mechanisms of p53-dependent apoptosis and proliferative inhibition in the regulation of abnormal ribosome biogenesis in ASMCs. Stress such as hypoxia that probably affects the RNA polymerase I or rRNA processing will result in the decrease of ribosome biosynthesis. In that case, the crucial proteins related to the muscle contraction were decreased. The decrease of “contractile unit” will lead to the impairment of the aortic wall. These abnormal ASMCs cannot fulfill its biological effects of antagonizing blood flow impact. Upon stimulation by the blood pressure, the impaired ASMCs would increase ROS production and trigger p53-dependent apoptosis process.

In total, we conceived a possible mechanism that was shown as a diagram (Figure 7). Stress such as hypoxia that probably affects the RNA polymerase I or rRNA processing will result in the decrease of ribosome biosynthesis. In that case, the crucial proteins related to the muscle contraction were decreased. The decrease of “contractile unit” will lead to the impairment of the aortic wall. These abnormal ASMCs cannot fulfill its biological effects of antagonizing blood flow impact. Upon stimulation by the blood pressure, the impaired ASMCs would increase ROS production and trigger p53-dependent apoptosis process. That might be one of the possible mechanisms that underline the AD.

## 4. Discussion

Under physiological conditions, the ASMCs need to constantly synthesize contractile proteins in order to maintain the stability of the aortic wall and cope with the powerful impact of blood flow [5]. The elastin-contractile unit is a functional and structural unit in the aortic media, which provides a direct connection between the ASMCs and the elastic fibres. The contractile unit in ASMCs is composed of thin filaments and thick filaments. The thick filament is composed of a smooth muscle-specific isoform of myosin heavy chain dimer (SM-MHC; encoded by MYH11) and four light chains (MLC), two regulatory light chains and two essential light chains. The thin filament is aggregated by *α*-SMA. Any reason that result in a decrease of contractile unit or its function will destroy the stability of the aortic wall [28]. In thoracic AD patients, over 60% of the DNA in ASMCs is hypermethylated indicating lower transcriptional activity and protein translation [29]. The number of ribosomes in a cell is closely related to its protein output, and ribosomal biogenesis and function can be disrupted by deregulated BOP1 expression [30], as well as inhibition of DNA methyltransferase activity [31]. BOP1 was significantly decreased in the aortic tissues of AD patients, and its knockdown in HASMCs impaired cell motility and decreased protein synthesis, as well as the expression of contraction-related proteins like *α*-SMA and MLC. This result is consistent with the higher susceptibility of individuals harbouring mutations in contraction-associated genes to AD [28, 32].

The phenotypic modulation of ASMCs from stable contractile cells to secretary proliferative cells is the major underlying mechanism of AMD [33, 34]. Microarrays of aortic tissues from AD patients (GEO: GSE52093) indicated increased expression of Ki-67 and PCNA [35, 36]. Contradictory to this observation, however, ASMC numbers generally decrease instead of increasing during AD [37, 38], which could be related to the higher apoptosis rates [39]. In our study, overexpressing BOP1 in HASMCs inhibited proliferation. This is consistent with the findings of Bornkamm et al. who showed that BOP1 expression alone cannot contribute to a fully functional PeBoW complex [40]. Interestingly, serum-free and hypoxic conditions downregulated BOP1 in a time-dependent manner and induced apoptosis, while overexpression of BOP1 inhibited this hypoxia-induced apoptosis and decreased contractile protein levels. Unlike Pes1 and WDR12, the two other proteins of the PeBoW complex, BOP1 has a short half-life on account of its extremely high PEST domain (common peptide motif of rapidly degrading proteins) score of 15.6 [41, 42]. Also, unlike its companion proteins, the expression of BOP1 in colon cancer cells is independent of c-myc activity [14]. Therefore, we hypothesize that the persistently high expression of exogenous BOP1 under hypoxic conditions may compensate for the PeBoW dysfunction caused by the rapid degradation of endogenous BOP1.

In order to impair ribosomal renewal in ASMCs *in vivo*, we treated the AD mice with cx-5461, an inhibitor of rRNA Pol I [43]. cx-5461 accelerated the occurrence of AD, inhibited the proliferation of ASMCs, and induced apoptosis. In a recent study, Ye et al. reported that cx-5461 prevented aortic intima hyperplasia, indicating its clinical potential against atherosclerosis and stenosis [44]. In contrast to our study, however, they showed that cx-5461 only inhibited ASMC proliferation and did not induce apoptosis [43]. Nevertheless, other reports have suggested that cx-5461 is capable of inducing tumor cell apoptosis [45–47]. The different results could be due to the different animal models used in these studies. We induced AD using BAPN, which inhibits the cross-linking of elastic fibres and weakens the structural toughness of the aorta [48]. This in turn results in severe stress on the ASMCs from the blood flow, leading to cellular degeneration and apoptosis.

The cell cycle arrest and apoptosis caused by ribosomal dysregulation are closely related to p53 [46, 47, 49], which is consistent with our results. Depletion of p53 by PFT*α* partially rescued the cx-5461-induced apoptosis *in vitro*. There are two possible mechanisms that can explain the association between p53 and ribosomal dysfunction. First, the reduction in rRNAs impairs ribosomal assembly, leading to an increase in free ribosomal proteins like ribosomal protein L (RPL) 11, RPL5, and RPL23, which can bind directly to MDM2 [50, 51]. This impedes MDM2-mediated ubiquitination of p53, resulting in apoptosis. The second model considers the mature ribosome as a “truck” that can transport the MDM2-p53 complex out of the nucleus for further degradation [52]. If the number of “trucks” is reduced, p53 accumulates in the nucleus and triggers its downstream proapoptotic signaling. To confirm whether p53-dependent apoptosis is the major cause of ASMC loss in AD, we established the AD model in p53-/- mice. As expected, the p53-/- AD mice survived longer and had lower rates of AD compared to the p53+/+ mice, possibly on account of enhanced proliferation and reduced apoptosis in the ASMCs.

However, knocking out p53 did not alleviate collagen accumulation and elastin breakdown *in vivo*. Almost all the mice that were fed with the BAPN diet eventually died. The AD animal model used in this study was different to the angiotensin II base mouse AD model, which was conducted by the pumping of angiotensin II in ApoE(-/-) mouse. BAPN inhibits cross-linking of elastic fibres and impairs the vascular structure, which increases the susceptibility of the ASMCs to vascular pressure. Furthermore, the structural impairment of elastic fibres decreases the anchoring of the transforming growth factor- (TGF-) *β*1 and suppresses TGF-*β*1 signaling in the ASMCs [53]. All these factors ultimately destroy the aorta and cause rapid death.

One limitation of our study was the imbalance in the age and gender distribution between the AD patients and donors, with significantly younger individuals and more males among the latter. The age bias was due to the fact that brain dead patients over the age of 50 are not considered as organ donors in China, and the gender bias is due to the fact that most organ donors are men. Another shortcoming of this study was that the effect of cx-5461 on p53-/- AD mice could not be evaluated due to the extremely low proportion (2-3%) of the p53-/- offspring produced by crossing p53+/- mice, a phenomenon consistent with Jackson Laboratory's description.

In conclusion, impaired ribosome biogenesis in the ASMCs accelerates cellular loss and leads to AD, a phenomenon that can be attenuated by p53 suppression. Ribosome biogenesis is under investigation as a novel target to treat cancer and intima hyperplasia. In light of our findings, however, the side-effects of targeting ribosome biogenesis and function, especially in patients with high risk of AD, should be strongly considered.

## Figures and Tables

**Table 1:** Clinical characters of the patients enrolled in this study.

	AD group (*n* = 28)	Donor group (*n* = 14)	*P* value
Male	19/28	13/14	<0.05
Age (mean ± SD)	51.46 ± 11.72	40.71 ± 10.64	<0.05
Hypertension	21/28	6/14	0.403
Type 2 diabetes	1/28	2/14	0.240
Liver complications	10/28	7/14	0.370
Renal complication	11/28	4/14	0.384

## Data Availability

The data used to support the findings of this study are available from the corresponding author upon request.

## Supplementary Materials

Supplementary Materials Fig. S1: PFT*α* inhibited superoxide production induced by cx-5461. HASMCs were pretreated with or without PFT*α* (10 *μ*M) for 12 h, followed by treatment of cx-5461 (1 *μ*M) for 24 h. The dihydroethidium (DHE) staining was used to detect ROS production (red). Fig. S2: inhibition of RNA polymerase I by cx-5461 promoted the accumulation of ROS in mouse AD model. The 8-OHdG level in mouse aortic medial tissues was detected by performing immunofluorescence and the representative images were shown. Scale bar 200 *μ*m. Fig. S3: knockout of p53 decreased the ROS accumulation in mouse AD model. The 8-OHdG level in mouse aortic medial tissues was detected by performing immunofluorescence and the representative images were shown. Scale bar 200 *μ*m.
