# Supplementary material for: Abnormal Ribosome Biogenesis Partly Induced p53-Dependent Aortic Medial Smooth Muscle Cell Apoptosis and Oxidative Stress
Source: Oxid Med Cell Longev. 2019 May 9;2019:7064319. doi: 10.1155/2019/7064319 (PMC6532287; doi:10.1155/2019/7064319)
Supplement: Supplementary Materials — Fig. S1: PFTα inhibited superoxide production induced by cx-5461. HASMCs were pretreated with or without PFTα (10 μM) for 12 h, followed by treatment of cx-5461 (1 μM) for 24 h. The dihydroethidium (DHE) staining was used to detect ROS production (red). Fig. S2: inhibition of RNA polymerase I by cx-5461 promoted the accumulation of ROS in mouse AD model. The 8-OHdG level in mouse aortic medial tissues was detected by performing immunofluorescence and the representative images were shown. Scale bar 200 μm. Fig. S3: knockout of p53 decreased the ROS accumulation in mouse AD model. The 8-OHdG level in mouse aortic medial tissues was detected by performing immunofluorescence and the representative images were shown. Scale bar 200 μm. [file 7064319.f1.pdf]

Supplementary Materials

Fig.S1 PFTα inhibited superoxide production induced by cx-5461.

HASMCs were pretreated with or without PFTα (10μM) for 12h, followed with treatment of cx-5461 (1μM) for 24h. The Dihydroethidium (DHE) staining was used to detect ROS production (red).

Fig.S2 Inhibition of RNA polymerase I by cx-5461 promoted the accumulation of ROS in mouse AD model.

The 8-OHdG level in mouse aortic medial tissues was detected by performing immunofluorescence and the representative images were shown. Scale bar 200μm.

Fig.S3 Knockout of p53 decreased the ROS accumulation in mouse AD model.

The 8-OHdG level in mouse aortic medial tissues was detected by performing immunofluorescence and the representative images were shown. Scale bar 200μm.

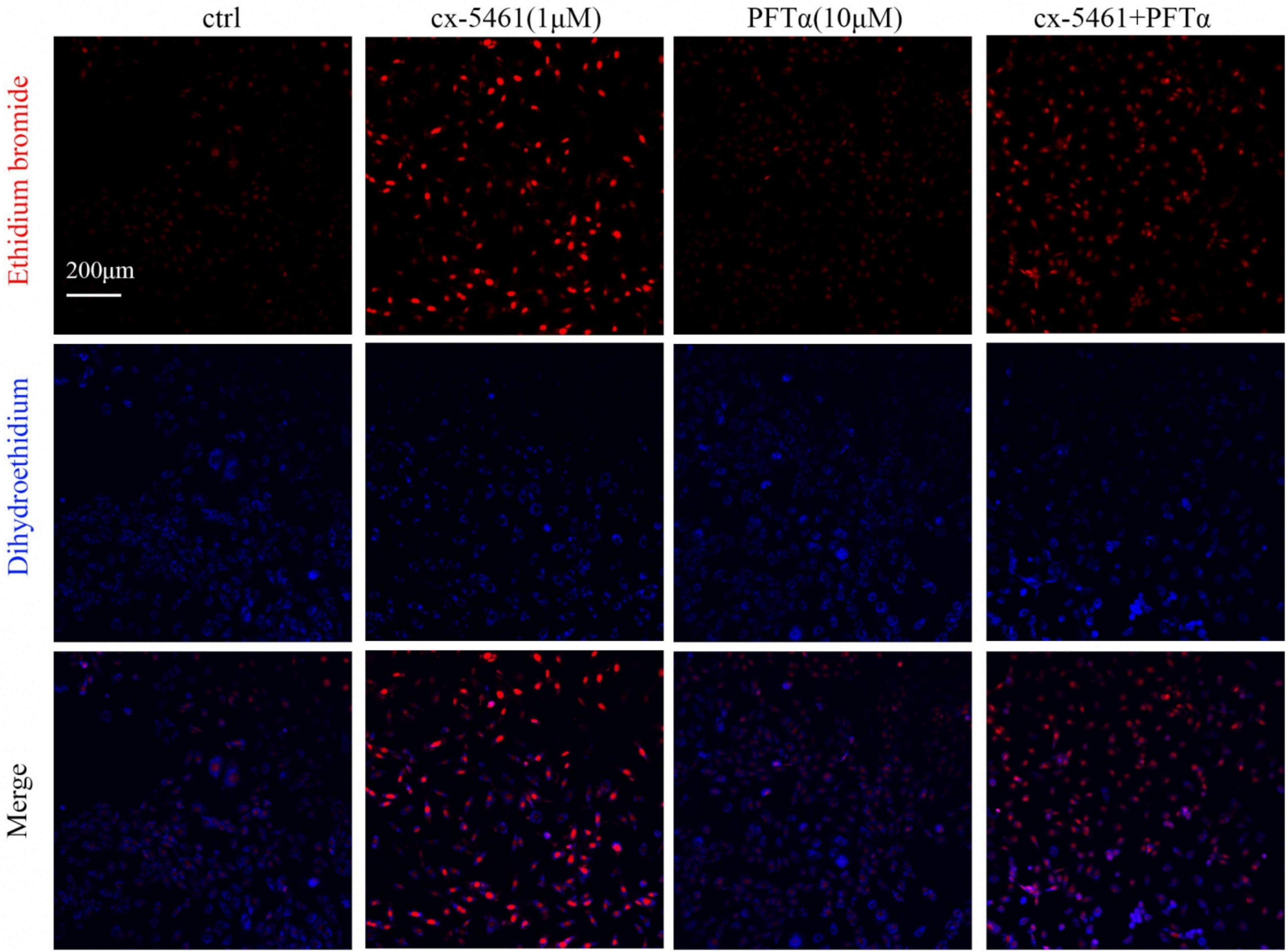

ctrl
cx-5461(1μM)
PFTα(10μM)
cx-5461+PFTα
Ethidium bromide
Dihydroethidium
Merge
200μm

Figure S1

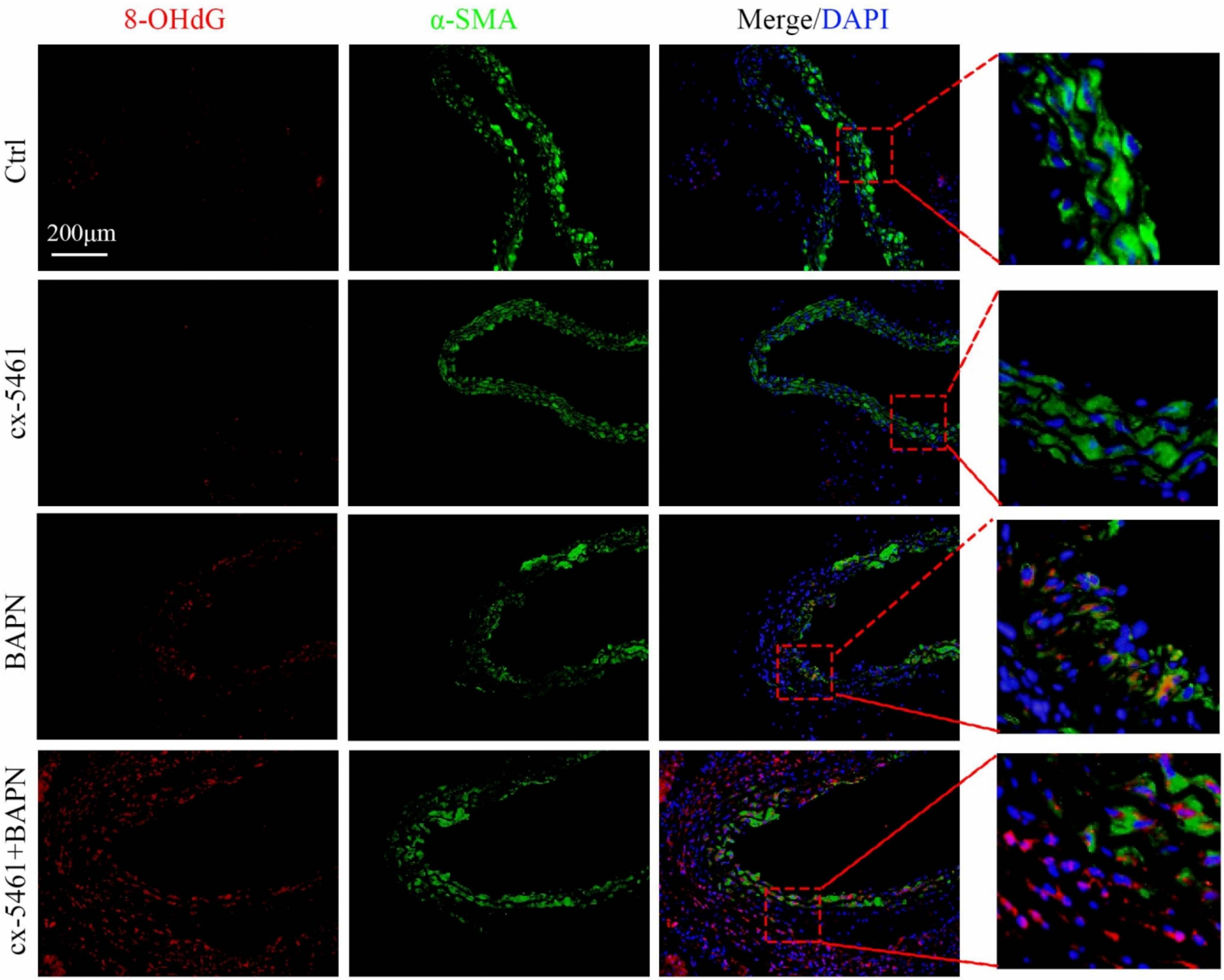

Figure S2

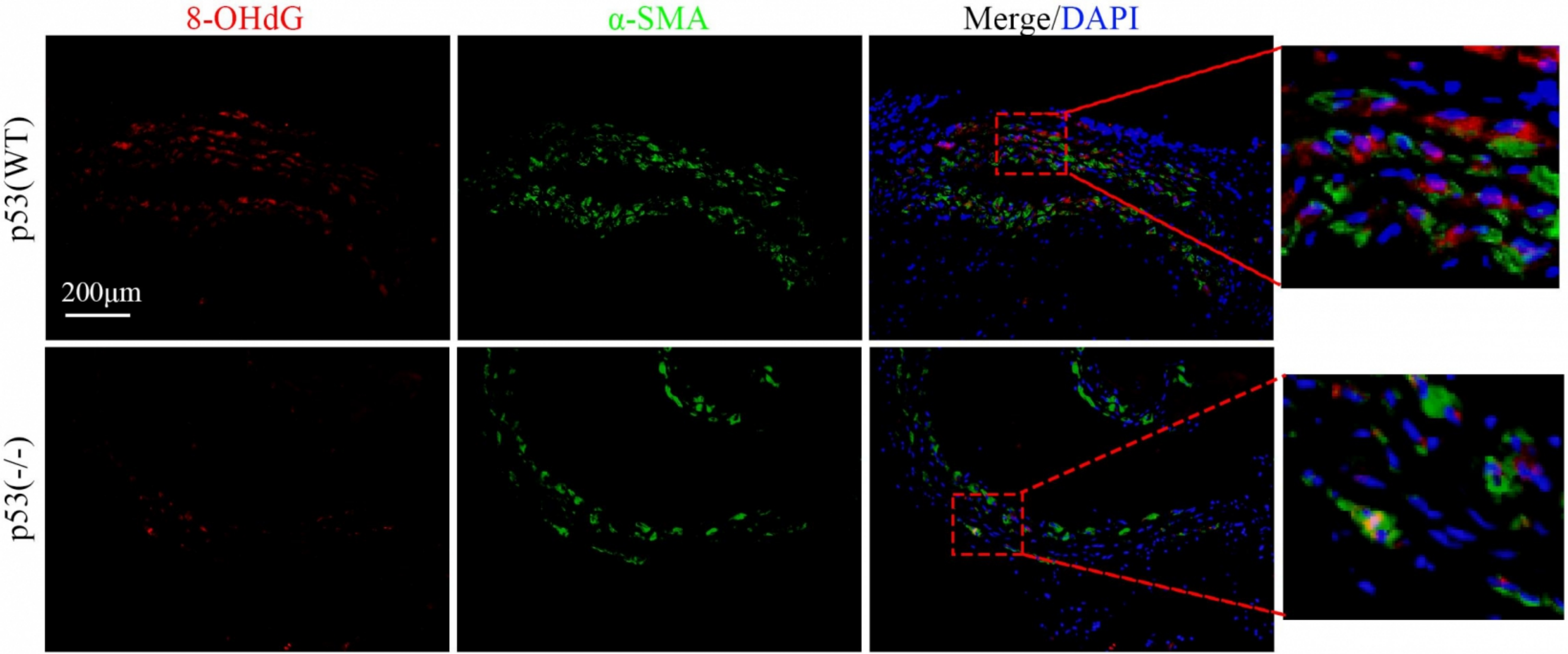

Figure S3
